# TREM2 promotes natural killer cell development in CD3^−^CD122^+^NK1.1^+^ pNK cells

**DOI:** 10.1186/s12865-021-00420-0

**Published:** 2021-05-12

**Authors:** Hwa-Youn Lee, Eun-Hee Lee, Jawoon Yi, Kon-Young Ji, Su-Man Kim, Ha-Rim Choi, Su-Min Yee, Hyung-Sik Kang, Eun-Mi Kim

**Affiliations:** 1 Chemicals Registration & Evaluation Team, National Institute of Environmental Research, Hwangyeong-ro 42, Seo-gu, Incheon, 22689 Korea; 2 Medical Device Development Center, Daegu-Gyeongbuk Medical Innovation Foundation, 80 Cheombok-ro, Dong-gu, Daegu, 41061 Korea; 3 School of Biological Sciences and Technology, Chonnam National University, 77 Yongbong-ro, Buk-gu, Gwangju, 61186 Republic of Korea; 4 Herbal Medicine Research Division, Korea Institute of Oriental Medicine, 461-24 Jeonmin-dong, Yuseong-gu, Daejeon, 34054 Korea; 5 Department of Nursing, Nambu University, 23 Chumdan Jungang-ro, Gwangsan-gu, Gwangju, 62271 Korea; 6 Department of Predictive Toxicology, Korea Institute of Toxicology, 141 Gajeong-ro, Yuseong-gu, Daejeon, 34114 Republic of Korea

**Keywords:** TREM2, Natural killer cell, NK cell development, Cancer immunotherapy

## Abstract

**Background:**

Triggering receptor expressed on myeloid cells 2 (TREM2) signaling is considered to regulate anti-inflammatory responses in macrophages, dendritic cell maturation, osteoclast development, induction of obesity, and Alzheimer’s disease pathogenesis. However, little is known regarding the effect of TREM2 on natural killer (NK) cells.

**Results:**

Here, we demonstrated for the first time that CD3^−^CD122^+^NK1.1^+^ precursor NK (pNK) cells expressed TREM2 and their population increased in TREM2-overexpressing transgenic (TREM2-TG) mice compared with that in female C57BL/6 J wild type (WT) mice. Both NK cell-activating receptors and NK cell-associated genes were expressed at higher levels in various tissues of TREM2-TG mice than in WT mice. In addition, bone marrow-derived hematopoietic stem cells (HSCs) of TREM2-TG mice (TG-HSCs) successfully differentiated into NK cells in vitro, with a higher yield from TG-HSCs than from WT-HSCs. In contrast, TREM2 signaling inhibition by TREM2-Ig or a phosphatidylinositol 3-kinase (PI3K) inhibitor affected the expression of the NK cell receptor repertoire and decreased the expression levels of NK cell-associated genes, resulting in significant impairment of NK cell differentiation. Moreover, in melanoma-bearing WT mice, injection of bone marrow cells from TREM2-TG mice exerted greater antitumor effects than that with cells from WT control mice.

**Conclusions:**

Collectively, our data clearly showed that TREM2 promoted NK cell development and tumor regression, suggesting TREM2 as a new candidate for cancer immunotherapy.

**Supplementary Information:**

The online version contains supplementary material available at 10.1186/s12865-021-00420-0.

## Background

Natural killer (NK) cells play key roles in both innate and adaptive immunity [1] by killing target cells through various mechanisms, including exocytosis of perforin and granzyme molecules, promotion of death receptor-mediated apoptosis, and secretion of interferon (IFN)-γ [2, 3]. The function of NK cells is regulated by a series of balancing signals derived from its activating and inhibitory receptors that interact with their respective ligands on target cells, including virus-infected, tumor, and allogeneic cells [4]. The ligand profile of the target cells allow NK cells to distinguish between self and non-self, as well as between normal and abnormal cells. Signals from NK cells that cause activation of receptors, such as NKG2C, NKG2D, NKG2E, Ly49C, Ly49D, Ly49H, and Ly49E (in mice), lead to cytotoxicity and cytokine production via signaling through DAP10 or DAP12 [5, 6]. In particular, the NKG2D/DAP10 receptor complex conveys activating signals by recruiting the p85 subunit of phosphatidylinositol 3-kinase (PI3K), leading to NK cell-mediated cytotoxicity [7, 8]. In contrast, engagement of Ly49A, Ly49C, Ly49G2, Ly49I, and NKG2A inhibitory receptors by major histocompatibility complex (MHC) class I hinders cytotoxicity and cytokine release [7, 8]. Additionally, the triggering receptor expressed on myeloid cells 2 (TREM2) is associated with DAP12 protein, which contains an immunoreceptor tyrosine-based activation motif (ITAM) that is structurally similar to NK cell receptors.

NK cells are derived from hematopoietic stem cells (HSCs) in the bone marrow (BM), fetal thymus, fetal liver, and umbilical cord blood [9–12]. In vitro culture of HSCs in medium supplemented with the stem cell factors FLT3L and interleukin (IL)-7 (defined as c-kit^+^ lineage^−^ HSCs) leads to their differentiation into precursor NK (pNK) cells that express CD122 receptor (IL-2Rβ^+^/CD15Rβ^+^) [12, 13]. These in vitro-differentiated CD122^+^ pNK cells further differentiate into immature NK cells (NK1.1^+^, CD49^−^NKG2^+^, and Ly49^−^) and mature NK (mNK) cells (NK1.1^+^, CD49/NKG2^+^, and Ly49^+^), when co-cultured with OP9 stromal cells in the presence of IL-2 or IL-15 [12–16]. Moreover, OP9 stromal cells secrete growth factors that support NK cell differentiation in vitro. We and other groups have previously reported gene expression profiles specific to NK cell differentiation in vitro, and we found that among these genes, inhibitor of DNA binding 2 (Id2) is an important transcription factor for NK cell differentiation [17]. In addition, E4bp4, which acts downstream of IL-15 receptor signaling, is an essential transcription factor for NK cell development and function [18]. Engagement of the IL-15 receptor with its ligand mediates NK cell activation [19] and differentiation [20] through the PI3K-protein kinase B (AKT) pathway. Additionally, we identified *TREM2* in the CD122^+^ pNK cell gene expression profile [10].

TREM2 is associated with DAP12, which contains the ITAM that serves as a docking site for Src kinases in dendritic cells (DCs) [7], osteoclasts [8], monocytes [21, 22], macrophages [23, 24], and microglia [21]. TREM2/DAP12 signaling induces PI3K and extracellular signal-regulated kinases expression [25], promotes the upregulation of CC chemokine receptor 7 in DCs [7], and increases phagocytosis in DCs [26, 27]. In addition, TREM2/DAP12 signaling negatively regulates the inflammatory response in microglia [27] and macrophages [28]. TREM2 undergoes intramembranous proteolysis by γ-secretase, while its extracellular domain is cleaved and removed by sheddase, disintegrin, and metalloproteinase domain-containing protein 10 [29, 30]. However, it is unclear whether the soluble and C-terminal fragments produced by this proteolytic event function as scavenger receptors or play biological roles [31, 32]. Moreover, TREM2/DAP12 has been shown to induce obesity by promoting adipogenesis and upregulating the expression of adipogenic regulators within adipocytes via WNT10b/β-catenin signaling [33]. Recently, TREM2 expression has also been identified as a risk factor for late-onset dementia and Alzheimer’s disease [34–39]. Furthermore, TREM2 can acts as a tumor suppressor in colorectal carcinoma and hepatocellular carcinoma through WNT1/β-catenin and extracellular signal-regulated kinase signaling or PI3K/AKT/β-catenin signaling [40, 41]. Nonetheless, although the structure of TREM2 is similar to that of NK cell receptors, its effect on NK cells remains unknown.

Here, we demonstrated that overexpression of TREM2 promoted NK cell differentiation and enhanced their cytotoxicity toward tumor cells in vivo and in vitro. Conversely, treatment with TREM2-Ig or a PI3K inhibitor inhibited NK cell differentiation, suggesting that activation of the PI3K pathway by TREM2/DAP12 signaling plays a crucial role in both the differentiation and effector function of NK cells.

## Results

### NK cell populations are increased in TREM2-overexpressing transgenic (TREM2-TG) mice

**Fig. 1:**
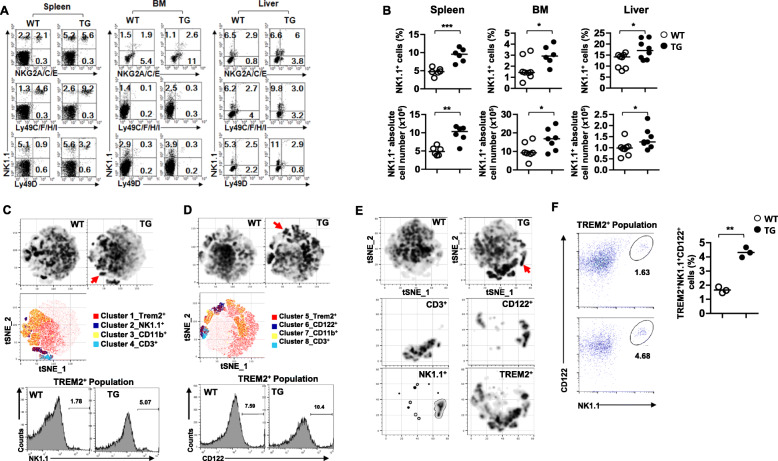
NK cell population increase in TREM2-TG mice. **a** Flow cytometry analysis data represent the surface expression of NK-specific receptors (NGK2A/C/E, Ly49C/F/H/I, Ly49D) in the spleen, BM, and liver of WT and TREM2-TG mice. **b** The percentage (top panel) and absolute number (bottom panel) of NK1.1^+^ cells in the spleen (*N* = 6), BM (*N* = 7), and liver (*N* = 8) of WT and TREM2-TG mice. Each symbol represents an individual mouse; horizontal lines indicate the mean. *P < 0.05, **P < 0.01 and ***P < 0.001 by Student’s t-test. **c**-**e** Representative tSNE plots show BM cell population of WT (left panel) and TG mice (middle panel) stained with TREM2/NK1.1/CD11b/CD3/TREM2 (**c**) or TREM2/CD122^+^/CD11b/CD3 specific antibodies (**d**). Each cluster represents a group of TREM2^+^ (Cluster1, 5), CD11b^+^ (Cluster 3,7), CD3^+^ (Cluster4, 8), NK1.1^+^ (Cluster2), and CD122^+^ (Cluster 6) cell population, respectively (**c** and **d** right panel). (**c**, right panel) Cluster2 (blue) represents NK1.1^+^ cells and cluster1 (red) represents TREM2+ cells (**d**, right panel). Red circles indicate the TREM2^+^ population on the NK1.1^+^ cells (**c**) or CD122^+^ cells (**d**). The histogram represents the TREM2^+^ population on the NK1.1^+^ cells (**c**, bottom) and TREM2^+^ population on the CD122^+^ cells (**d**, bottom) of TG and WT mice. **e** These tSNE plots showed CD3^+^, CD122^+^, NK1.1^+^, and TREM2^+^ cell population in the BMs of WT (left panel) and TG mice (right panel). Red circles indicate the CD3^−^CD122^+^NK1.1^+^TREM2^+^ population in BMs of TG mice. Each tSNE plot represents CD3^+^, CD122^+^, NK1.1^+^, TREM2^+^ cell population separately. **f** Dot plots represent that BM of TG mice (left, lower) and WT mice (left, upper), which were stained with TREM2+, CD122+, and NK1.1+ antibodies. Black circles indicate CD122 + NK1.1+ population in the TREM2+ population. The percentage (right) of TREM2+ CD122 + NK1.1+ cells in the BM (*N* = 3) of WT and TREM2-TG mice. tSNE plots and dot plots were analyzed using Flow jo software

To investigate the effect of TREM2 on NK cell development, we analyzed NK cell populations in previously generated TREM2-overexpressing transgenic (TREM2-TG) and wild type (WT) mice using flow cytometry [33]. The expression of NK receptor repertoires, NK1.1+ population percentage, and their absolute numbers in the spleen, BM, and liver were higher in TREM2-TG mice than in WT (Fig. 1a and b). Furthermore, the absolute number of NK cells expressing the NKG2A/NKG2C/NKG2E receptor was higher in the spleens (Additional file 1; Fig. S1, left panel) and livers (right panel) of TREM2-TG mice than that in those of WT mice. A slight increase in the BM of TREM2-TG mice (middle panel) was also observed. Similarly, the percentage and absolute number of Ly49C/F/H/I^+^ and Ly49D^+^ NK cells in the spleens and BMs of TREM2-TG mice were significantly higher than in those of WT (Fig. 1b and Additional file 1, Fig. S1).

To investigate whether NK cells express TREM2, we isolated BM cells from WT and TREM2-TG mice, stained them with NK-specific markers, and performed flow cytometry analysis. The t-SNE density plots of the BM cells obtained from WT and TREM2-TG mice are shown in Fig. 1c–e left and middle panels, respectively. The BM cells analyzed expressed both NK1.1^+^ (Cluster 2, blue) and TREM2 surface protein (red) (Fig. 1c, third panel). Furthermore, NK1.1^+^ TREM2^+^ (blue and red) population was increased in the BMs of TREM2-TG mice (5.07%) (Fig. 1c, red arrow and histogram) compared to the WT (1.78%). Similarly, BM cells expressed CD122^+^ (Cluster 6, blue) and TREM2+ (red) (Fig. 1d, third panel), and an increase in CD122^+^ TREM2^+^ double-positive (blue and red) cells was observed in TREM2-TG mice (10.4%) (red arrow and histogram) compared to WT mice (7.59%). Then, we further analyzed TREM2 expression in pNK cells (CD3^−^ CD122^+^ NK1.1^+^). As shown in Fig. 1e, TREM2-expressing pNK cells were increased in TREM2-TG mice compared with WT mice. In addition, the frequency of TREM2+ pNK cell population (TREM2^+^ CD122^+^ NK1.1^+^) was significantly higher in the BMs of TG mice (4.68%) than in that of WT mice (1.63%) (Fig. 1f). These data showed that TREM2 was expressed in pNK cells.

Reverse transcription-polymerase chain reaction (RT-PCR) was performed to identify whether TREM2 regulates NK cell function-associated genes using splenic NK1.1^+^ cells from WT and TREM2-TG mice. The expression levels of *Ifng* (IFN-γ) (3.29 ± 1.158-fold increase), *Prf1* (perforin) (18.25 ± 5.3-fold increase), and *Gzmb* (granzyme B) (57 ± 16.97-fold increase) were significantly higher in the spleens of TREM2-TG mice than those of WT mice (Additional file 1, Fig. S2A). Consequently, TREM2-TG splenic NK cells showed significantly higher NK cell-mediated cytotoxicity than those of WT mice (Additional file 1, Fig. S2B). No differences were observed between the frequency and absolute number of CD4^+^, CD8^+^, and B220^+^ cells in the spleens of WT and TREM2-TG mice (Additional file 1, Fig. S3).

### In vivo inhibition of TREM2 signaling reduces NK cell populations

**Fig. 2:**
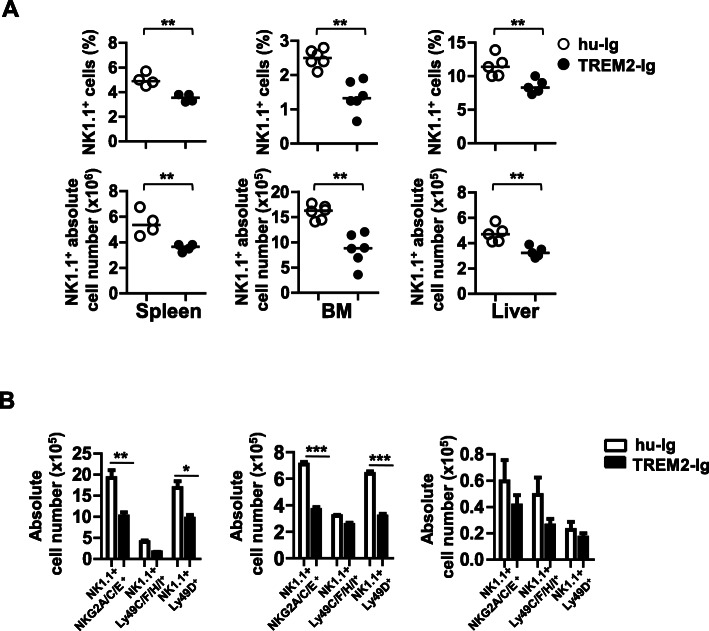
Blockade of TREM2 signaling reduces the NK cell population in vivo. **a** WT mice were injected with 100 μg of TREM2-Ig or hu-Ig (control) twice per week for 4 weeks intraperitoneally, then the expression of NK cell-specific receptors in the spleen, BM, and liver cells of WT mice was analyzed using flow cytometry. **a** The percentage (top panel) and absolute number (bottom panel) of NK1.1^+^ cells from the spleen (*N* = 4), BM (N = 6), and liver (*N* = 5) of mice as shown in Fig. 1a. Each symbol represents an individual mouse; horizontal lines indicate the mean. **P < 0.01 by unpaired Student’s t-test. **b** Graphs of absolute cell number of NK cells expressing each NK cell receptor in the spleen (left panel), BM (middle panel), and liver (right panel) of mice treated with hu-Ig (open bar) or TREM2-Ig (solid bar). For each population, the absolute number determined by calculation from flow cytometry profiles. Data are shown as mean ± SEM from three independent experiments. *P < 0.05, **P < 0.01 and ***P < 0.001 by based on two-way ANOVA analysis with Bonferroni posthoc test

To verify the effect of TREM2 on NK cell development in vivo, we inhibited TREM2 signaling in WT mice via intraperitoneal injection of a TREM2-Ig fusion protein or a humanized (hu)-Ig control. Three days after injection, the spleen, BM, and liver cells were isolated, and NK cell populations and NK-specific receptors expression were analyzed by flow cytometry. The frequency and absolute number of NK1.1^+^ cells, in the total splenocyte population, were reduced in the spleens of TREM2-Ig-injected mice, compared to hu-Ig-injected control mice (3.525% ± 0.32% vs. 5% ± 0.5%; Fig. 2a and Additional file 1, Fig. S4). Similarly, the percentage and absolute number of NK1.1^+^ cells in the BM and liver of TREM2-Ig-injected mice were lower than in hu-Ig-injected control mice (Fig. 2a). Furthermore, in the spleen, the percentage of NK1.1^+^ NKG2A/C/E^+^ cells was lower in TREM2-Ig-injected mice (1.9%) than in hu-Ig-injected control mice (2.4%) (Additional file 1, Fig. S4, left panel). Both the NK1.1^+^ NKG2A/C/E^+^ (0.6% vs. 1.1%) and NK1.1^+^ Ly49D^+^ (0.5% vs. 1%) populations were reduced in the BMs of TREM2-Ig-injected mice when compared with the control group; the absolute number of NK1.1^+^ cells was also decreased by TREM2 signaling inhibition (Fig. 2b and Additional file 1, Fig. S4, middle panel). In addition, the frequency of NK1.1^+^ NKG2A/C/E^+^ (4.9% vs. 6.3%) and NK1.1^+^ Ly49D^+^ (2% vs. 2.4%) populations in the liver, as well as the absolute number of NK1.1^+^ cells, was lower in TREM2-Ig-injected mice than in control mice (Fig. 2b and Additional file 1, Fig. S4, right panel). However, the absolute numbers of NK1.1^+^ NKA/C/E^+^, Ly49C/F/H/I^+^, and Ly49D^+^ cells were significantly decreased in the spleens (Fig. 2b, left panel) and BMs (Fig. 2b, middle panel), but not in the livers (Fig. 2b, right panel), of TREM-Ig-injected mice, when compared with those values found in the hu-Ig-injected mice (Fig. 2b, right panel). These data collectively indicate that in vivo inhibition of TREM2 signaling by TREM2-Ig decreases the number of NK cells and the expression of their signature surface receptors.

### TREM2 promotes NK cell differentiation and direct cytotoxic activity in vitro

**Fig. 3:**
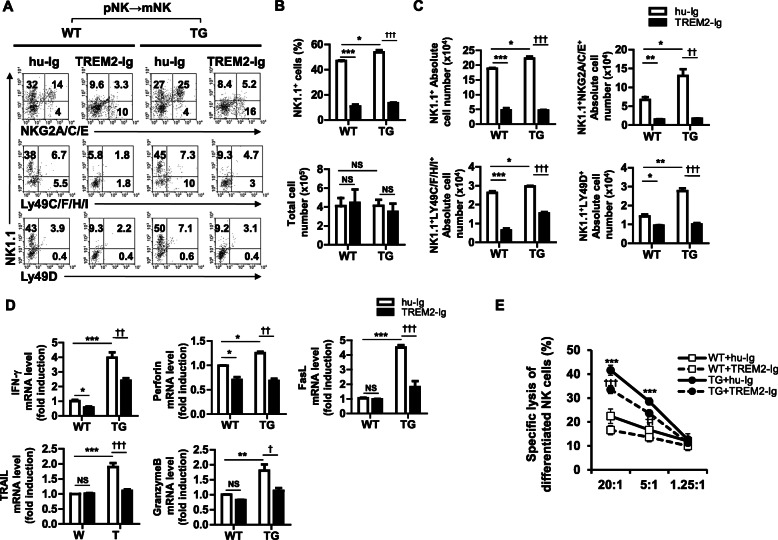
Effects of TREM2 on the differentiation of NK cells from HSCs in vitro. **a** Representative flow cytometry plots of NK cell receptor expression on the differentiated NK cells from WT-HSCs or TREM2-TG-HSCs. Cells treated with hu-Ig or TREM2-Ig during the pNK to mNK cell differentiation simultaneously. **b** Graphs indicate the total number of cells (upper panel) and percentage of NK1.1^+^ cells (lower panel) differentiated from WT or TG –HSCs, where were treated with hu-Ig (open bar) or TREM2-Ig (solid bar). **c** Graphs show the absolute cell number of the NK1.1^+^ cell population, NK receptor-expressing NK cells that were differentiated from WT-HSCs treated with hu-Ig (open bar) or TREM2-Ig (solid bar). **d** Real-time-qPCR analysis of IFN-γ, Perforin, Granzyme B, FasL, and TRAIL mRNAs in mNK cells derived from WT-HSCs or TG-HSCs. Values are presented as the mean ± standard error of the mean of three independent experiments (**b**-**d**). **e** Cytotoxic effect of differentiated NK cells derived from WT-HSCs and TREM2-HSCs treated with hu-Ig or TREM2-Ig since the pNK stage. *P < 0.05, **P < 0.01, ***P < 0.001 vs. WT-hu-Ig and †P < 0.05, ††P < 0.01, and †††P < 0.001 vs. TG-hu-Ig by two-way ANOVA analysis with Bonferroni posthoc test. Three independent experiments were performed

Our results showed that TREM2 signaling increased the number of NK cells in vivo. However, this is not sufficient to conclude that TREM2 enhances commitment to the NK cell fate and differentiation of the NK cell lineage. Therefore, to determine the effects of TREM2 on the differentiation of NK cells, we isolated c-kit^+^ Lin^−^ HSCs from BMs of WT and TREM2-TG mice and differentiated them into pNK and mNK cells in vitro. During NK cell differentiation, pNK cells were treated with TREM2-Ig or hu-Ig to inhibit TREM2 signaling. As a result, the percentage and absolute numbers of NK1.1^+^ NKG2A/C/E^+^ cells were approximately 2-fold higher in the mNK cells derived from hu-Ig-treated pNK cells of TREM2-TG mice (25%) than in their counterparts derived from hu-Ig-treated pNK cells of WT mice (14%) (Fig. 3a). However, the NK1.1^+^ NKG2A/C/E^+^ cell population dramatically decreased when WT-pNK (46.8% ± 0.8 to 10.6% ± 2.7%) and TREM2-TG-pNK (53.8% ± 2.8 to 13.3% ± 0.88%) cells were treated with TREM2-Ig during differentiation (Fig. 3a and b, upper panel). The absolute number of NK1.1^+^ cells decreased by 3.97- (WT) and 4.77-folds (TREM2-TG) after treatment with TREM2-Ig (Fig. 3c). Additionally, the percentage and absolute number of NK cells expressing either Ly49C/F/H/I or Ly49D were reduced in both NK cells derived from WT-pNK (6.7 to 1.8% and 3.9 to 2.2%, respectively) and TREM2-pNK (7.3 to 4.7% and 7.1 to 3.1%, respectively) after TREM2-Ig treatment during differentiation (Fig. 3a and c). In contrast, the difference between the total number of cells differentiated from TREM2-TG-pNK or WT-pNK cells was not significative, regardless of TREM2-Ig treatment (Fig. 3b, lower panel). Subsequently, we performed RT-PCR analyses to identify the expression of NK cell-associated genes that are regulated by TREM2 (Fig. 3d and Additional file 1, Fig. S5). NK cells that differentiated from TREM2-TG-pNK cells treated with hu-Ig showed increased *Ifng* (3.97 ± 0.63-fold) and Fas ligand (*Faslg*) (4.5 ± 0.3-fold) expression compared with those NK cells derived from WT-pNK hu-Ig-treated cells. We also observed increased expression levels of *Gzmb* (1.8 ± 0.36-fold), *Prf1* (1.25 ± 0.05-fold), and TNF-related apoptosis-inducing ligand (*Trail*) (1.9 ± 0.22-fold) in NK cells derived from TREM2-TG-pNK cells treated with hu-Ig, when compared with those derived from WT-pNK hu-Ig treated cells. In contrast, the expression levels of these genes were reduced in NK cells derived from both WT-pNK and TG-pNK cells, when TREM2 signaling was inhibited by TREM2-Ig (Fig. 3d). Moreover, the expression levels of *E4bp4*, *Id2*, *CD122*, and *CD123* increased in TREM2-TG-pNK-derived NK cells when compared to those in WT-pNK-derived NK cells treated with hu-Ig, while in differentiated NK cells, it decreased significantly after TREM2-Ig treatment (Additional file 1, Fig. S5). Next, to elucidate the effect of TREM2 on the cytotoxicity of differentiated NK cells, we performed an in vitro NK cell cytotoxicity assay. The mNK cells differentiated from TREM2 TG-pNK treated with TREM2-Ig or hu-Ig showed significantly higher specific cytolytic activity (32 ± 1.2% and 41 ± 2.4%, respectively) against target cells at a 20:1 (Effector: Target) ratio (Fig. 3e) than those differentiated from WT-pNK cells treated with hu-Ig. At a 5:1 ratio, we observed that NK cells differentiated from TREM2 TG-pNK hu-Ig or TREM2-Ig treated cells showed a significative higher level of target cell death than cells from WT-pNK that received the same treatments (Fig. 3e). Therefore, these data suggest that TREM2 signaling increases in vitro cytotoxic activity of differentiated NK cells.

### TREM2 signaling inhibits tumor progression

**Fig. 4:**
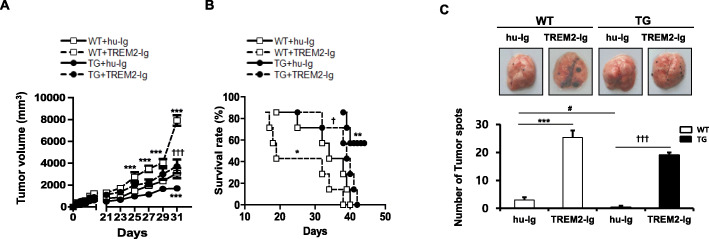
TREM2 signaling prevents tumor progression in vivo. **a** Tumor volumes of WT and TREM2-TG mice. WT and TREM2-TG Mice were injected with B16-F10 cells subcutaneously after intraperitoneal injection of hu-Ig or TREM2-Ig. Tumor volumes were measured every 2 days. **b** The survival rate of tumor-bearing WT and TREM2-TG mice. Seven mice per group were used. Survival probabilities were analyzed using the Kaplan–Meier method. The significance of differences between groups assessed using the log-rank test. All statistical tests were two-sided, with *P < 0.05 taken to indicate significance. Significance of difference between samples was determined using two-way ANOVA analysis with Bonferroni posthoc test. WT + hu-Ig vs. TG + hu-Ig (**P < 0.01), WT + hu-Ig vs. WT + TREM2-Ig (*P < 0.05), and TG + hu-Ig vs. TG + TREM2-Ig (†P < 0.05, †††P < 0.001) were compared. **c** Representative photographs of lungs with metastatic colonies from mice of each group (top panel). Graph quantitates the total number of metastatic colonies in the lungs of each group treated with hu-Ig or TREM2-Ig (bottom panel). WT + hu-Ig vs. TG + hu-Ig (^#^P < 0.01) WT + hu-Ig vs. WT + TREM2-Ig (***P < 0.05), and TG + hu-Ig vs. TG + TREM2-Ig (†††P < 0.001) were compared. Significance of difference between samples was determined using two-way ANOVA analysis with Bonferroni posthoc test

As described above, the inhibition of TREM2 signaling pathway by TREM2-Ig reduced NK cell receptor and NK cell-associated gene expression, as well as the absolute number of NK cells in vitro (Fig. 3). To confirm whether TREM2 affects tumor progression in vivo, we injected TREM2-TG or WT mice with B16F10 melanoma cells after intraperitoneal injection of hu-Ig or TREM2-Ig. As shown in Fig. 4a, on day 25, the tumor volume in WT mice treated with TREM2-Ig (WT + TREM2-Ig) was significantly higher than that in WT mice treated with hu-Ig (WT + hu-Ig), and these differences became even more prominent after day 25. Additionally, on day 25, the tumor volume in TREM2-TG mice treated with TREM2-Ig (TG + TREM2-Ig) was higher (1988.14 ± 426.2 mm^3)^ than that in TREM2-TG mice treated with hu-Ig (TG + hu-Ig) (970.3 ± 257.11 mm^3^). Furthermore, on day 31, tumor volume was significantly lower in TG + hu-Ig mice (1700.82 ± 171.142 mm^3^) than in WT + hu-Ig mice 3088.09 ± 808.67 mm^3^). On average, TREM2-TG mice tumor volume was approximately 2-fold lower (3718.48 ± 1095.74 mm^3^) than in WT mice (7915.32 ± 839.09 mm^3^). These data suggested that tumor progression in TREM2-TG mice was significantly reduced compared with WT mice, and tumor progression in both TREM2-TG and WT mice increased upon TREM2-Ig treatment. In addition, differences in survival rate were observed upon inhibition of TREM2 signaling. The survival rate of tumor-bearing WT mice injected with TREM2-Ig was 14.2%, which was significantly lower than that of tumor-bearing WT mice injected with hu-Ig (42.8%), tumor-bearing TREM2-TG mice injected with TREM-Ig (71.4%), and tumor-bearing TREM2-TG mice injected with hu-Ig (100%) on day 38 (Fig. 4b). Furthermore, the number of metastatic melanomas in the lungs of TREM2-Ig-injected WT mice was higher (24 ± 4, B16F10 cell spots) than that of hu-Ig-injected WT mice (3 ± 1, B16F12 cell spots) (Fig. 4c). Surprisingly, B16F10 melanoma cells were rarely observed in the lungs of hu-Ig-injected TREM2-TG mice, whereas melanoma cells were apparent in the lungs of TREM2-Ig-injected TREM2-TG mice (19 ± 1, B16F10 cell spots).

### Adoptive transfer of TREM2-TG BM cells promotes tumor regression

**Fig. 5:**
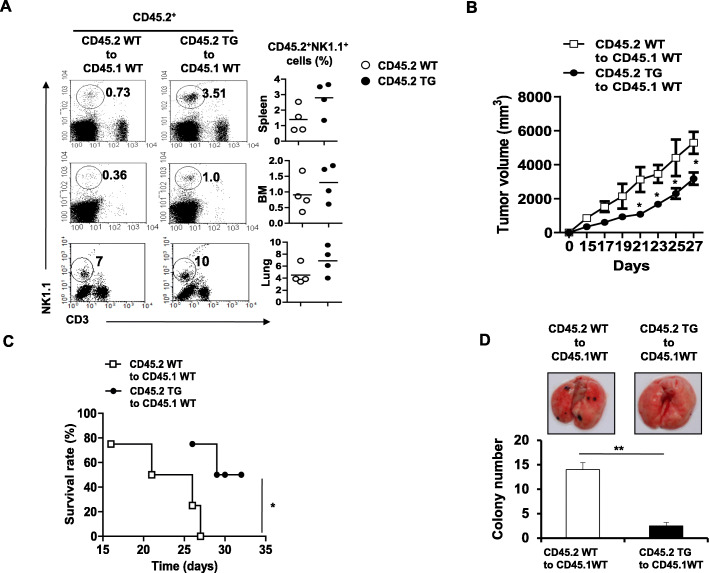
Adoptive bone marrow transplantation of TREM2 TG mice increase NK cell population in WT mice. **a** Representative flow cytometry plots of CD3^−^NK1.1^+^cells from spleen, BM, and lung of lethally irradiated WT CD45.1 recipient 4 weeks after tail vein injection of total BM from WT or TREM2-TG (CD45.2) donors. The percentage (left) of CD45.2 + NK1.1+ cells in the spleen, BM, and lung of WT CD45.1 recipient (*N* = 4). Each symbol represents an individual mouse; horizontal lines indicate the mean. **b** Graph depicting tumor volumes in tumor-bearing CD45.1 WT mice after intravenous injection with BM cells of CD45.2 WT (open square, N = 4) or CD45.2 TREM2-TG mice (solid circle, N = 4). Tumor volume was measured every 2 days, after 15 days of injection. Significance of difference was analyzed by two-way ANOVA analysis with Bonferroni posthoc test (*P < 0.05). Three independent experiments repeated. **c** The survival rate of tumor-bearing CD45.1 WT mice after intravenous injection with BM cells from CD45.2 WT (open square, N = 4) or CD45.2 TREM2-TG mice (solid circle, N = 4). *P < 0.05. **d** Representative photographs of lungs with metastatic colonies (top panel) of tumor-bearing CD45.1 WT mice after intravenous injection with BM cells of CD45.2 WT or CD45.2 TREM2-TG mice. Graph quantifies the total number of metastatic colonies in the lung (bottom panel). **P < 0.01 using Student’s t-test

As mentioned above, TREM2-TG mice showed a significantly lower tumor volume and rare metastatic tumor spots compared with WT mice when they were injected with B16F10 melanoma cells. This may be related to the effects of TREM2-overexpressing monocytes/macrophages or DCs, which secrete cytokines and indirectly activate T cells and NK cells. To investigate whether TREM2-overexpressing BM-derived immune cells can cause tumor regression, we transplanted CD45.2 TG-BMs (TG to WT) or CD45.2 WT-BMs (WT to WT) into sub-lethally irradiated WT recipients (CD45.1). Four weeks after adoptive transfer, we analyzed the NK cell population in various organs of each group by flow cytometry. We detected higher proportions of NK cells (NK1.1+ CD3– CD45.2+) in the spleens (3.51% vs. 0.73%), BMs (1.0% vs. 0.36%), and lungs (10% vs. 7%) of CD45.1 WT recipients engrafted with CD45.2 TG-BMs than in those with CD45.2 WT-BMs (Fig. 5a). These data indicated that TREM2-TG mice have a larger NK cell population than WT mice.

Then, we used an in vivo tumor model to determine whether TREM2 signaling affects the antitumor effect of BM-derived immune cells. We subcutaneously injected B16-F10 melanoma cells into WT mice (CD45.1) engrafted with BMs (CD45.2) from WT or TREM2-TG mice and measured the tumor volume every other day. The tumor volume measured 21 days post-inoculation in mice transplanted with a TREM2-TG-BM was lower (TG to WT, 1079 ± 221.5 mm3) than that of mice that received a WT-BM (WT to WT controls, 3122.7 ± 1269 mm3) (Fig. 5b). Furthermore, 27 days post-inoculation, the survival rate (75%) of tumor-bearing mice transplanted with a TREM2-TG-BM was significantly higher than that of WT-BM-transplanted mice (0%) (Fig. 5c). To observe lung metastatic melanoma, we sacrificed mice from each group on day 14. Several melanomas (large black spots, 14 ± 1) were observed in the lung tissues of WT-BM-recipients (open bars, Fig. 5d); however, only a few melanomas were detectable (2 ± 1) in lung tissue of TREM2-TG-transplanted mice (solid bars, Fig. 5d). Moreover, CD45.2^+^ NK1.1^+^ cells persisted in tumor-bearing mice transplanted with a TREM-2-TG-BM at day 41 post adaptive transplantation (Fig. S9).

### TREM2 regulates NK cell differentiation via PI3K signaling

TREM2-DAP12 signaling, triggered by TREM2 ligand binding, may promote or inhibit proinflammatory responses, induce obesity [33], and mediate neurodegeneration [34, 38]. DAP12, an adaptor protein of TREM2, mediates downstream signaling via the cytoplasmic ITAM domain, which recruits SYK and activates PI3K, phospholipase C, and Vav signaling cascades [42]. To investigate how TREM2 signaling regulates NK cell differentiation, we treated pNK cells differentiated from WT-HSCs or TG-HSCs with the PI3K inhibitor Ly294002 or with dimethyl sulfoxide (DMSO) as control vehicle during their differentiation into mNK cells. After 14 days, differentiated mNK cells were stained with NK-specific markers and analyzed via flow cytometry (Additional file 1, Fig. S6A). In the absence of the PI3K inhibitor, population of NK1.1^+^ NKG2ACE^+^ cells differentiated from TREM2-TG-pNK cells Additional file 1, Fig. S6A, lower panel) was 2-fold higher than that of NK cells differentiated from WT-pNK cells (Fig. S6A, upper panel). NK1.1^+^ NKG2ACE^+^ cell populations derived from both WT- and TREM2-TG-pNK cells decreased (10-fold) after Ly294002 treatment during NK cell maturation.

We also analyzed the expression of NK cell-associated genes in mNK cells differentiated in the presence or absence of PI3K inhibitor. The expression levels of *Ifng*, *Prf1*, and *Gzmb* increased by 4- to 5-fold in mNK cells differentiated from TREM2-TG-pNK cells compared to mNK cells differentiated from WT-HSCs. Similarly, the expression levels of *Faslg*, *Trail*, and IL-15Rα were higher in mNK cells differentiated from TREM2-TG-pNK cells than those in cells differentiated from WT-pNK cells. With the exception of *E4bp4* and IL-15Rα, NK cell-related gene expression levels significantly decreased in mNK cells treated with Ly49294002 during NK cell differentiation (Additional file 1, Fig. S6). In particular, the expression level of *Id2* decreased by more than 2-fold in mNK cells after treatment with Ly294002.

## Discussion

TREMs have emerged as critical immune regulators that modulate inflammatory responses in macrophages, glial cells, and DCs [41, 43–45]. Recently, several groups reported TREM2 as a novel tumor suppressor in colorectal and hepatocellular carcinoma [40, 46]. However, the function of TREM2 in NK cells has not yet been elucidated. NK cells mediate innate as well as adaptive immune responses, including anti-tumoral cytotoxic activity; nevertheless, autologous NK therapy for patients with cancer has shown several limitations [47], such as difficulties in NK cell expansion ex vivo and the severe side effects observed after IL-2 treatment for NK cell activation [40]. Therefore, it is necessary to find new ways to regulate NK cell function. It is known that NK cells are modulated by their activating receptors, which present binding motifs for the adaptor protein DAP12. The structure of TREM2 is largely similar to that of other NK cell receptors that transmit intracellular signals via DAP12, although its function in NK cells remains unclear.

In this study, we found that TREM2 is expressed in CD3^−^ CD122^+^ NK1.1^+^ pNK cells and that NK cell population abundance in the BMs of TREM2-TG mice was higher than that in the BMs of WT mice. It was recently reported that liver lymphocytes express DAP12, as well as low levels of TREM2; this information supports the findings of the current study [48].

The late NK cell maturation stage (CD3^−^NK1.1^+^) can be subdivided into four distinct subsets: CD27^lo^ Mac-1^lo^, CD27^hi^ Mac-1^lo^, CD27^hi^ Mac-1^hi^, and CD27^lo^ Mac-1^hi^ [49–51]. The intermediate CD27^hi^ Mac-1^hi^ population shows the strongest cytotoxicity and cytokine secretion [52], and it has a higher proliferation potential and an enhanced ability to interact with DCs [51]. Conversely, CD27^lo^ Mac-1^hi^ NK cells, the most abundant mNK cells, are effective killer cells in vivo, and are particularly effective against MHC class I-negative tumor cells [49]. According to our data, TREM2 overexpression affected CD27 ^hi^Mac-1^lo^, CD27^hi^ Mac-1^hi^, and CD27^lo^ Mac-1^hi^ populations in the BM, suggesting that TREM2 promoted NK cell maturation in the BM with strong expression of TREM2 ligands (Additional file 1, Fig. S7). In contrast, in the peripheral blood, the number of NK1.1^+^ CD3^−^ cells was higher in TREM2-TG mice (8.2%) than in WT mice (5.7%), whereas NK1.1^+^ CD3^−^ CD27^hi^ Mac-1^hi^ cell population presence was similar in the two groups of mice (Additional file 1, Fig. S7B). These data suggested that TREM2 did not affect NK cell maturation in the peripheral blood, although more NK cells were released into the blood in TREM2-TG mice, as NK cell maturation was promoted in the BMs of TREM2-TG mice.

In addition, we established a tumor-bearing mouse model to demonstrate that the NK cell population increased via TREM2 overexpression, which reduced tumor progression. The tumor volume in tumor-bearing WT mice transplanted with BMs from TREM2-TG mice was lower than that in WT mice transplanted with BMs from WT mice. The metastasis of tumor cells in the lung tissue was reduced, and the survival rate of WT mice transplanted with BMs from TREM2-TG mice was higher than that of mice transplanted with BMs from WT mice. NK cells promote the maturation of DCs via IFN-γ, an important proinflammatory cytokine [53], while DCs stimulate NK cytotoxicity and cytokine secretion via IL-12 [54]. This bidirectional crosstalk between NK cells and DCs is an important mechanism in innate and adaptive immune responses [55, 56]. Therefore, TREM2 reduces tumor progression in vivo by directly improving NK cell cytotoxicity, and TREM2-overexpressing DCs and macrophages could develop and stimulate NK cell function in vivo.

We co-cultured pNK cells derived from WT-HSCs and TG-HSCs with OP9 stromal cells, which support hematopoiesis by secreting growth factors, to induce further differentiation into mNK cells. Bartosz et al. [57] reported that NK cells may be derived from myeloid progenitors. Thus, we hypothesized that TREM2 in pNK cells or myeloid progenitors enhanced both the differentiation of NK cells in vitro and their cytotoxicity in the presence of OP9 cells. Recently, apolipoprotein E has been reported as a ligand of TREM2, although this is now considered controversial [58]. Consequently, the identification of TREM2 ligands is still necessary in order to develop NK cell therapies.

Previous studies have demonstrated that PI3K, and not PLC-γ, plays a critical role in the development of mNK cells [59]; moreover, while the absence of PLC-γ does not disrupt NK cell development, it causes defects in NK cell cytotoxicity [60, 61]. However, Tassi et al. [62] have demonstrated that PLC-γ2 is crucial for the development of the NK cell receptor repertoire. In the current study, expression of the NKG2A/NKG2C/NKG2E receptor in mNK cells, differentiated from WT-pNK or TREM2-TG-pNK cells, decreased significantly from 40.68 to 8.96% after PLC-γ inhibitor treatment (Additional file 1, Fig. S8A). The expression of *Id2* was upregulated by TREM2 in mNK cells differentiated from WT-pNK or TREM2-TG-pNK cells, and it was downregulated upon PI3K inhibitor treatment. Moreover, the expression of *E4bp4*, an essential factor for NK cell development [18], was upregulated by TREM2 in NK cells differentiated from pNK cells and was downregulated following PLC-γ inhibitor treatment (Additional file 1, Fig. S8B), indicating that TREM2 influenced NK cell differentiation not only via the PI3K signaling pathway but also via PLC-γ signaling.

## Conclusions

In conclusion, we demonstrated that TREM2 played an important role not only in myeloid cells but also in CD3^−^ CD122^+^ NK1.1^+^ pNK cells. Furthermore, TREM2 promoted NK cell differentiation, as well as the expression of NK cell receptor repertoires and cytokines, suggesting that TREM2 might be an effective candidate for new NK cell therapies.

## Methods

### Mice

Five to seven-week-old female C57BL/6 J WT and TREM2-TG mice were used in this study as described previously [33]. The TREM2-TG mice were generated using pcDNA3.1(+) expression vector containing pCMV promoter. Overexpression of the TREM2 gene was observed in all tissues. All animal experiments were carried out following the guidelines of the Institutional Animal Care Committee of Chonnam National University (CNU IACUC-YB-2017-19).

### Differentiation of HSCs into mNK cells

Murine HSCs were sorted from BM cell populations by negative or positive selection using a magnetic-activated cell sorter (MACS), as described previously [10]. Briefly, total BM cell samples were prepared by flushing the femurs from C57BL/6 mice, followed by filtration through a 70-μm cell strainer (Falcon, San Jose, CA, USA). Total BM samples were cleared of erythrocytes with Erythrocyte Lysis Buffer (Sigma-Aldrich, St. Louis, MO, USA) treatment. The suspensions of single BM cells were labeled using a cocktail of biotinylated antibodies against lineage (Lin^+^) markers (CD11b, Gr-1, B220, NK1.1, CD2, and TER-119), which were then incubated on streptavidin-magnetic beads. The samples were depleted of magnetically labeled Lin^+^ cells by retention on CS column beads in the magnetic field of a VarioMACS Separator (Miltenyi Biotec, Sunnyvale, CA, USA). c-Kit^+^ cells among the Lin^−^ cells were positively selected with magnetic bead-conjugated antibodies against c-kit, and then, the cell suspension was run through an MS magnetic column Separator (Miltenyi Biotec, Sunnyvale, CA, USA).

HSCs were stimulated to differentiate into NK cells, as described previously [11]. In brief, purified Lin^−^ c-kit^+^ HSCs were plated on a 24-well plate (Corning, ME, USA) at 1 × 10^6^ cells/well and cultured in RPMI medium supplemented with a mixture of IL-7 (0.5 ng/mL), stem cell factor (30 ng/mL), Flt3-L (50 ng/mL), indomethacin (20 μg/mL), and gentamycin (20 μg/mL) at 37 °C and 5% CO_2_. Three days later, half of the culture supernatant was removed and replaced with fresh medium containing the same cytokines. After 7 days, the cells were co-cultured with or without OP9 stromal cells (American Type Culture Collection, Manassas, VA, USA) in the presence of mouse IL-15 (20 ng/mL). Three days later, half of the culture medium was changed with fresh medium containing the same cytokines, and the cells were cultured for an additional 7 days. To determine purity, the cells were stained on days 0, 7, and 14 with stage-specific antibodies during the differentiation of NK cells and analyzed via flow cytometry.

### Isolation of NK1.1^+^ cells from the spleen

Splenocytes were isolated from the spleens of the mice, and the cell suspension was filtered through a 20-μm cell strainer. After removal of erythrocytes via treatment with erythrocyte lysis buffer (Sigma-Aldrich, St. Louis, MO, USA), the single cells in suspension were first incubated with a biotinylated antibody against NK1.1 (BD Pharmingen, San Diego, CA, USA), followed by incubation with streptavidin-magnetic beads. NK1.1^+^ cells were then purified using MACS (Miltenyi Biotec) according to the manufacturer’s instructions.

### Flow cytometry analysis

To determine the developmental status of NK cells differentiated from HSCs of WT and TREM2-TG mice, we performed flow cytometry analysis of HSCs, pNKs, and mNKs co-cultured with OP9 cells using antibodies against the markers with stage-specific expression during the differentiation of NK cells. In brief, HSCs were stained with 1 μL of fluorescein isothiocyanate (FITC)-conjugated anti-c-kit, phycoerythrin (PE)-conjugated anti- Sca-1, and biotin/streptavidin/cytochrome-conjugated anti-IL-7Rα. pNKs were stained with 1 μL of FITC-conjugated anti-CD122 and 0.5 μL of PE-conjugated anti-NK1.1 antibodies. mNKs were stained with FITC-conjugated anti-NKG2A/C/E, anti-Ly49C/F/H/I, and anti-Ly49D and PE-conjugated anti-NK1.1 antibodies. The cells were incubated with the antibodies for 30 min on ice and then washed twice with staining buffer (phosphate-buffered saline containing 3% fetal bovine serum and 0.1% NaN_3_). The cells were analyzed using a FACS Calibur flow cytometer (BD Bioscience, San Jose, CA, USA) and Cell Quest software. The data shown in the histograms or dot plots are representative of replicates.

### BM adoptive transfer

WT (CD45.1) recipients were irradiated with 6.5 Gy, followed by injection with WT (CD45.2) or TREM2-TG (CD45.2) BM cells (1 × 10^6^) intravenously. Four to eight weeks after the cells were transplanted, the BM and spleen were harvested, and single-cell suspensions were prepared as described above. Erythrocytes were lysed, lymphoid cell populations were counted, and the number of NK cells was assessed using flow cytometry with antibodies against NK1.1, CD45.1, or CD45.2.

### Tumor models

To determine the tumor volume and survival rate, we subcutaneously injected B16F10 melanoma cells (5 × 10^5^ cells/mouse) (ATCC, VA, USA) into the left flank of WT mice, TREM2-TG mice, BM-transplanted WT mice, and mice intraperitoneally injected with TREM2-Ig or hu-Ig (as a control). After B16F10 cell injection, the tumor volume was measured every 2 days.

### RT-PCR

Total cellular RNA was extracted using Trizol B reagent (Tel-Test, Friendswood, TX, USA) according to the manufacturer’s instructions. Aliquots of total RNA were transcribed into cDNA at 37 °C for 1 h in a total reaction volume of 20 μL with 2.5 U of Moloney murine leukemia virus reverse transcriptase (Roche, Mannheim, Germany). Reverse-transcribed cDNA was added to a PCR mixture consisting of 10× PCR buffer, 0.2 mM dNTP, 0.5 U Taq DNA polymerase (Bioneer, Daejeon, Korea), and 10 pmol of primers for each gene. For *β-actin* amplification, 27 cycles were performed, and for all other genes, 30 or 35 cycles were performed. The amplification profile included denaturation at 95 °C for 1 min, primer annealing at 55 °C for 1 min, and extension at 72 °C for 10 min. PCR products were electrophoresed and visualized via ethidium bromide staining.

### Cytotoxicity assay

The lactate dehydrogenase-release assay kit (Promega, WI, USA) was used to measure the cytotoxicity of NK cells, according to the manufacturer’s instructions. In brief, NK cells were stimulated with 20 ng/mL of recombinant murine IL-2 for 48 h, washed twice with phosphate-buffered saline, and seeded into 96-well round-bottom microtiter tissue culture plates at various effector:target cell ratios. Target cell samples (1 × 10^4^ cells per well) were tested in triplicate. The cells were incubated for 4 h at 37 °C in a 5% CO_2_ humidified incubator. Culture supernatants (50 μL) were then collected and combined with 50 μL of the substrate. The plates were covered with aluminum foil for protection against light and incubated at room temperature for 30 min, after which 50 μL of stop solution was added to each well. Absorbance at 490 nm was measured within 1 h of adding the stop solution. The results are expressed as the percentage of specific release based on the following formula: percent specific release = [(experimental release - spontaneous release)/(maximum release - spontaneous release)] × 100.

### Statistical analysis

All values are expressed as mean ± standard error of the mean (SEM). All experiments were repeated at least three times, independently. Student’s *t*-test and analysis of variance were performed using GraphPad Prism 5 (San Diego, CA, USA). Differences were considered statistically significant at *P* < 0.05.

## Supplementary Information

**Additional file 1: Fig. S1.** The number of NK1.1^+^ cells, from TREM2-TG mice, expressing NK cell receptors is higher than in WT mice. Graphs show the absolute number of cells that express the NK cell receptors in the spleen, BM, and liver of WT (opened bar) and TREM2-TG mice (solid bar). Data are shown as mean ± SED of three independent experiments. *P < 0.05, **P < 0.01, and ***P < 0.001 by Student’s t-test. **Fig. S2.** TREM2-TG mice show enhanced NK cell cytotoxicity. (A) Real-time qPCR analysis to determine expression of the *TREM2*, *Ifng*, *Prf1*, and *G*zmb (granzyme B) mRNAs in splenic NK1.1^+^ cells of WT or TREM2-TG mice. (B) LDH assay to measure cytotoxicity of NK1.1^+^ cells purified from splenocytes harvested from WT (open square) and TREM2-TG (solid circle) mice. Three independent experiments were performed (A-B). *P < 0.05, **P < 0.01, and ***P < 0.001 by Student’s t-test. **Fig. S3.** CD4^+^T cell, CD8^+^T cell, and B220^+^ B cell frequency and absolute number in WT and TREM2-TG mice are similar. Percentage (A) and absolute number (B) of CD4, CD8, and B cells in spleen of WT and TREM2-TG mice determined by flow cytometry (*N* = 5). Three independent experiments were performed, but no significant differences were observed between WT and TG mice group. **Fig. S4.** Inhibition of TREM2 signaling reduces the NK cell pool in vivo. Representative flow cytometry plots of expression of NK cell-specific receptors (NKG2A/C/E, Ly49C/F/H/I, and Ly49D) on surface of cells isolated from the spleen, BM, and liver of WT mice injected (i.p.) with 100 μg of TREM2-Ig or hu-Ig (control) twice per week for 4 weeks. **Fig. S5**. TREM2 signaling enhances NK cell-related gene expression in differentiated NK cells in vitro. Quantitative real-time PCR analysis of NK cell-associated genes using mRNA isolated from mNK cells derived from WT or TREM2-TG HSCs. Data are shown as mean ± SED of three independent experiments. *P < 0.05, **P < 0.01, ***P < 0.001 vs. WT+ hu-Ig, and †P < 0.05, ††P < 0.01, †††P < 0.01 vs. TG + hu-Ig, based on two-way ANOVA with Bonferroni post-hoc test. **Fig. S6.** The development and cytotoxicity of NK cells is regulated by TREM2 via PI3K signaling. (A) Representative flow cytometry plots showing the relative ratio of mNK cells developed in vitro from pNK cells of WT-HSCs and TREM2-HSCs treated with DMSO or LY294002 (1 μM). (B) Quantitative real-time PCR analysis of the indicated genes using mRNA isolated from mNK cells differentiated from WT-HSCs or TREM2-TG-HSCs treated with DMSO (opened bar) or LY294002 (solid bar). *P < 0.05, **P < 0.01, ***P < 0.001 vs. WT+ DMSO, and †P < 0.05, ††P < 0.01 vs. TG + DMSO, based on two-way ANOVA with Bonferroni post-hoc test. **Fig. S7.** The CD3^−^NK1.1^+^Mac-1^+^CD27^+^ NK cell subset is reduced in peripheral blood of TREM2-K/O mice. Representative flow cytometry plots show the NK cell subsets (CD3^−^NK1.1^+^Mac-1^low^CD27^high^, CD3^−^NK1.1^+^Mac-1^high^ CD27_high_, CD3^−^NK1.1^+^Mac-1^high^ CD27^low^, and CD3^−^NK1.1^+^Mac-1^low^CD27^high^) in the peripheral blood (A) or BM (B) of WT and TREM2-TG mice. Three independent experiments were performed. **Fig. S8.** TREM2 regulates NK cell differentiation via PLC-γ signaling pathway. (A) Representative flow cytometry analysis showing NK1.1 and NKG2A/C/E expression on the differentiated NK cells derived from TG-HSCs treated with DMSO (control) or U-73122 (PLC-γ inhibitor) during pNK to mNK cell differentiation stage. (B) RT-PCR analysis of the indicated genes on mNK cells differentiated from WT-HSCs or TREM2 TG-HSCs treated with DMSO (control) or U-73122 (PLC-γ inhibitor). β-actin (housekeeping gene) was used as a control. The cropped images from the different parts of the gel were displayed. The full-length gels are presented in Fig. S9. **Fig. S9.** The CD45.2^+^NK1.1^+^ cells persist in the tumor site in the recipient mice that received TREM2-TG-BM transplantation. Representative flow cytometry analysis of the expression of CD45.2^+^ NK1.1^+^ cells derived from TREM2-TG-HSCs in the tumor model (*N* = 3). Data from day 41 after the 4 weeks adaptive transplantation following period. **Fig. S10.** Full-length gel images of cropped gels. (A) Full-length gel corresponding to Fig. S8B; E4bp4 (left), Id2 (middle), TREM2 (right). (B) Full-length gel corresponding to Fig. S8B, *β*-actin.

## Data Availability

Not applicable.

## Abbreviations

TREM2: Triggering receptor expressed on myeloid cells 2
NK: Natural killer
pNK: precursor natural killer
mNK: mature natural killer
TREM2-TG: TREM2-overexpressing transgenic
HSC: Hematopoietic stem cell
TG-HSC: Bone marrow-derived HSC of TREM2-TG mice
PI3K: Phosphatidylinositol 3-kinase
MHC: Major histocompatibility complex
ITAM: Immunoreceptor tyrosine-based activation motif
Id2: Inhibitor of DNA binding 2
RT-PCR: Reverse transcription-polymerase chain reaction
TRAIL: TNF-related apoptosis-inducing ligand
WT + TREM2-Ig: WT mice treated with TREM2-Ig
Hu: Humanized
WT + hu-Ig: WT mice treated with hu-Ig
TG + TREM2-Ig: TREM2-TG mice treated with TREM2-Ig
TG + hu-Ig: TREM2-TG mice treated with hu-Ig
DMSO: Dimethyl sulfoxide
MACS: Magnetic-activated cell sorter

## References

[1] Trinchieri G (1989). Biology of natural killer cells. Adv Immunol.

[2] Moretta A, Bottino C, Mingari MC, Biassoni R, Moretta L (2002). What is a natural killer cell?. Nat Immunol.

[3] Purdy AK, Campbell KS (2009). Natural killer cells and cancer: regulation by the killer cell Ig-like receptors (KIR). Cancer Biol Ther.

[4] Rajaram N, Tatake RJ, Advani SH, Gangal SG (1990). Natural killer and lymphokine activated killer cell functions in Hodgkin's disease. Br J Cancer.

[5] Bryson JS, Flanagan DL (2000). Role of natural killer cells in the development of graft-versus-host disease. J Hematother Stem Cell Res.

[6] Sternberg-Simon M, Brodin P, Pickman Y, Onfelt B, Karre K, Malmberg KJ, Hoglund P, Mehr R (2013). Natural killer cell inhibitory receptor expression in humans and mice: a closer look. Front Immunol.

[7] Bouchon A, Hernandez-Munain C, Cella M, Colonna M (2001). A DAP12-mediated pathway regulates expression of CC chemokine receptor 7 and maturation of human dendritic cells. J Exp Med.

[8] Paloneva J, Mandelin J, Kiialainen A, Bohling T, Prudlo J, Hakola P, Haltia M, Konttinen YT, Peltonen L (2003). DAP12/TREM2 deficiency results in impaired osteoclast differentiation and osteoporotic features. J Exp Med.

[9] Choi J, Hwang YK, Sung KW, Lee SH, Yoo KH, Jung HL, Koo HH, Kim HJ, Kang HJ, Shin HY, Ahn HS (2007). Expression of Livin, an antiapoptotic protein, is an independent favorable prognostic factor in childhood acute lymphoblastic leukemia. Blood.

[10] Kang HS, Kim EM, Lee S, Yoon SR, Kawamura T, Lee YC, Kim S, Myung PK, Wang SM, Choi I (2005). Stage-dependent gene expression profiles during natural killer cell development. Genomics.

[11] Lee KN, Kang HS, Jeon JH, Kim EM, Yoon SR, Song H, Lyu CY, Piao ZH, Kim SU, Han YH, Song SS, Lee YH, Song KS, Kim YM, Yu DY, Choi I (2005). VDUP1 is required for the development of natural killer cells. Immunity.

[12] Lian RH, Kumar V (2002). Murine natural killer cell progenitors and their requirements for development. Semin Immunol.

[13] Williams NS, Klem J, Puzanov IJ, Sivakumar PV, Bennett M, Kumar V (1999). Differentiation of NK1.1+, Ly49+ NK cells from flt3+ multipotent marrow progenitor cells. J Immunol.

[14] Caraux A, Lu Q, Fernandez N, Riou S, Di Santo JP, Raulet DH, Lemke G, Roth C (2006). Natural killer cell differentiation driven by Tyro3 receptor tyrosine kinases. Nat Immunol.

[15] Douagi I, Colucci F, Di Santo JP, Cumano A (2002). Identification of the earliest prethymic bipotent T/NK progenitor in murine fetal liver. Blood.

[16] Williams NS, Kubota A, Bennett M, Kumar V, Takei F (2000). Clonal analysis of NK cell development from bone marrow progenitors in vitro: orderly acquisition of receptor gene expression. Eur J Immunol.

[17] Zook EC, Li ZY, Xu Y, de Pooter RF, Verykokakis M, Beaulieu A, Lasorella A, Maienschein-Cline M, Sun JC, Sigvardsson M et al: Transcription factor ID2 prevents E proteins from enforcing a naive T lymphocyte gene program during NK cell development. Sci Immunol 2018;3(22):eaao2139. 10.1126/sciimmunol.aao2139 PMC6750718 29703840

[18] Gascoyne DM, Long E, Veiga-Fernandes H, de Boer J, Williams O, Seddon B, Coles M, Kioussis D, Brady HJ (2009). The basic leucine zipper transcription factor E4BP4 is essential for natural killer cell development. Nat Immunol.

[19] Ali AK, Nandagopal N, Lee S-H (2015). IL-15-PI3K-AKT-mTOR: a critical pathway in the life journey of natural killer cells. Front Immunol.

[20] Mace EM (2018). Phosphoinositide-3-kinase signaling in human natural killer cells: new insights from primary immunodeficiency. Front Immunol.

[21] Schmid CD, Sautkulis LN, Danielson PE, Cooper J, Hasel KW, Hilbush BS, Sutcliffe JG, Carson MJ (2002). Heterogeneous expression of the triggering receptor expressed on myeloid cells-2 on adult murine microglia. J Neurochem.

[22] Bouchon A, Dietrich J, Colonna M (2000). Cutting edge: inflammatory responses can be triggered by TREM-1, a novel receptor expressed on neutrophils and monocytes. J Immunol.

[23] Chung DH, Seaman WE, Daws MR (2002). Characterization of TREM-3, an activating receptor on mouse macrophages: definition of a family of single Ig domain receptors on mouse chromosome 17. Eur J Immunol.

[24] Gordon S, Taylor PR (2005). Monocyte and macrophage heterogeneity. Nat Rev Immunol.

[25] Neubauer A, Fiebeler A, Graham DK, O'Bryan JP, Schmidt CA, Barckow P, Serke S, Siegert W, Snodgrass HR, Huhn D (1994). Expression of axl, a transforming receptor tyrosine kinase, in normal and malignant hematopoiesis. Blood.

[26] Hsieh CL, Koike M, Spusta SC, Niemi EC, Yenari M, Nakamura MC, Seaman WE (2009). A role for TREM2 ligands in the phagocytosis of apoptotic neuronal cells by microglia. J Neurochem.

[27] Takahashi K, Rochford CD, Neumann H (2005). Clearance of apoptotic neurons without inflammation by microglial triggering receptor expressed on myeloid cells-2. J Exp Med.

[28] Turnbull IR, Gilfillan S, Cella M, Aoshi T, Miller M, Piccio L, Hernandez M, Colonna M (2006). Cutting edge: TREM-2 attenuates macrophage activation. J Immunol.

[29] Kleinberger G, Yamanishi Y, Suarez-Calvet M, Czirr E, Lohmann E, Cuyvers E, Struyfs H, Pettkus N, Wenninger-Weinzierl A, Mazaheri F (2014). TREM2 mutations implicated in neurodegeneration impair cell surface transport and phagocytosis. Sci Transl Med.

[30] Wunderlich P, Glebov K, Kemmerling N, Tien NT, Neumann H, Walter J (2013). Sequential proteolytic processing of the triggering receptor expressed on myeloid cells-2 (TREM2) protein by ectodomain shedding and gamma-secretase-dependent intramembranous cleavage. J Biol Chem.

[31] Colonna M, Wang Y (2016). TREM2 variants: new keys to decipher Alzheimer disease pathogenesis. Nat Rev Neurosci.

[32] Peng Q, Malhotra S, Torchia JA, Kerr WG, Coggeshall KM, Humphrey MB (2010). TREM2- and DAP12-dependent activation of PI3K requires DAP10 and is inhibited by SHIP1. Sci Signal.

[33] Park M, Yi JW, Kim EM, Yoon IJ, Lee EH, Lee HY, Ji KY, Lee KH, Jang JH, Oh SS, Yun CH, Kim SH, Lee KM, Song MG, Kim DH, Kang HS (2015). Triggering receptor expressed on myeloid cells 2 (TREM2) promotes adipogenesis and diet-induced obesity. Diabetes.

[34] Benitez BA, Jin SC, Guerreiro R, Graham R, Lord J, Harold D, Sims R, Lambert JC, Gibbs JR, Bras J (2014). Missense variant in TREML2 protects against Alzheimer's disease. Neurobiol Aging.

[35] Jiang T, Yu JT, Zhu XC, Tan L (2013). TREM2 in Alzheimer's disease. Mol Neurobiol.

[36] Jin SC, Carrasquillo MM, Benitez BA, Skorupa T, Carrell D, Patel D, Lincoln S, Krishnan S, Kachadoorian M, Reitz C, Mayeux R, Wingo TS, Lah JJ, Levey AI, Murrell J, Hendrie H, Foroud T, Graff-Radford NR, Goate AM, Cruchaga C, Ertekin-Taner N (2015). TREM2 is associated with increased risk for Alzheimer's disease in African Americans. Mol Neurodegener.

[37] Replogle JM, Chan G, White CC, Raj T, Winn PA, Evans DA, Sperling RA, Chibnik LB, Bradshaw EM, Schneider JA, Bennett DA, de Jager PL (2015). A TREM1 variant alters the accumulation of Alzheimer-related amyloid pathology. Ann Neurol.

[38] Walter J (2016). The triggering receptor expressed on myeloid cells 2: a molecular link of Neuroinflammation and neurodegenerative diseases. J Biol Chem.

[39] Zhong L, Chen XF, Zhang ZL, Wang Z, Shi XZ, Xu K, Zhang YW, Xu H, Bu G (2015). DAP12 stabilizes the C-terminal fragment of the triggering receptor expressed on myeloid Cells-2 (TREM2) and protects against LPS-induced pro-inflammatory response. J Biol Chem.

[40] Kim SM, Kim EM, Ji KY, Lee HY, Yee SM, Woo SM, Yi JW, Yun CH, Choi H, Kang HS (2019). TREM2 Acts as a Tumor Suppressor in Colorectal Carcinoma through Wnt1/beta-catenin and Erk Signaling. Cancers..

[41] Zheng H, Jia L, Liu C-C, Rong Z, Zhong L, Yang L, Chen X-F, Fryer JD, Wang X, Zhang Y-W, Xu H, Bu G (2017). TREM2 promotes microglial survival by activating Wnt/β-catenin pathway. J Neurosci.

[42] Colonna M (2003). TREMs in the immune system and beyond. Nat Rev Immunol.

[43] Klesney-Tait J, Turnbull IR, Colonna M (2006). The TREM receptor family and signal integration. Nat Immunol.

[44] Hamerman JA, Jarjoura JR, Humphrey MB, Nakamura MC, Seaman WE, Lanier LL (2006). Cutting edge: inhibition of TLR and FcR responses in macrophages by triggering receptor expressed on myeloid cells (TREM)-2 and DAP12. J Immunol.

[45] Cantoni C, Bollman B, Licastro D, Xie M, Mikesell R, Schmidt R, Yuede CM, Galimberti D, Olivecrona G, Klein RS, Cross AH, Otero K, Piccio L (2015). TREM2 regulates microglial cell activation in response to demyelination in vivo. Acta Neuropathol.

[46] Tang W, Lv B, Yang B, Chen Y, Yuan F, Ma L, Chen S, Zhang S, Xia J (2019). TREM2 acts as a tumor suppressor in hepatocellular carcinoma by targeting the PI3K/Akt/β-catenin pathway. Oncogenesis.

[47] Bachanova V, Miller JS (2014). NK cells in therapy of cancer. Crit Rev Oncog.

[48] Nakao T, Ono Y, Dai H, Nakano R, Perez-Gutierrez A, Camirand G, Huang H, Geller DA, Thomson AW (2019). DNAX activating protein of 12 kDa/triggering receptor expressed on myeloid cells 2 expression by mouse and human liver dendritic cells: functional implications and regulation of liver ischemia-reperfusion injury. Hepatology.

[49] Hayakawa Y, Smyth MJ (2006). CD27 dissects mature NK cells into two subsets with distinct responsiveness and migratory capacity. J Immunol.

[50] Pinhas N, Sternberg-Simon M, Chiossone L, Shahaf G, Walzer T, Vivier E, Mehr R (2016). Murine peripheral NK-cell populations originate from site-specific immature NK cells more than from BM-derived NK cells. Eur J Immunol.

[51] Watt SV, Andrews DM, Takeda K, Smyth MJ, Hayakawa Y (2008). IFN-gamma-dependent recruitment of mature CD27(high) NK cells to lymph nodes primed by dendritic cells. J Immunol.

[52] Wickström SL, Öberg L, Kärre K, Johansson MH (2014). A genetic defect in mice that impairs missing self recognition despite evidence for Normal maturation and MHC class I–dependent education of NK cells. J Immunol..

[53] Raulet DH, Guerra N (2009). Oncogenic stress sensed by the immune system: role of natural killer cell receptors. Nat Rev Immunol.

[54] Zwirner NW, Ziblat A (2017). Regulation of NK cell activation and effector functions by the IL-12 family of cytokines: the case of IL-27. Front Immunol.

[55] Harizi H (2013). Reciprocal crosstalk between dendritic cells and natural killer cells under the effects of PGE2 in immunity and immunopathology. Cell Mol Immunol.

[56] Walzer T, Dalod M, Robbins SH, Zitvogel L, Vivier E (2005). Natural-killer cells and dendritic cells: “l’union fait la force”. Blood.

[57] Grzywacz B, Kataria N, Kataria N, Blazar BR, Miller JS, Verneris MR (2011). Natural killer-cell differentiation by myeloid progenitors. Blood.

[58] Atagi Y, Liu CC, Painter MM, Chen XF, Verbeeck C, Zheng H, Li X, Rademakers R, Kang SS, Xu H, Younkin S, Das P, Fryer JD, Bu G (2015). Apolipoprotein E is a ligand for triggering receptor expressed on myeloid cells 2 (TREM2). J Biol Chem.

[59] Guo H, Samarakoon A, Vanhaesebroeck B, Malarkannan S (2008). The p110 delta of PI3K plays a critical role in NK cell terminal maturation and cytokine/chemokine generation. J Exp Med.

[60] Caraux A, Kim N, Bell SE, Zompi S, Ranson T, Lesjean-Pottier S, Garcia-Ojeda ME, Turner M, Colucci F (2006). Phospholipase C-gamma2 is essential for NK cell cytotoxicity and innate immunity to malignant and virally infected cells. Blood.

[61] Kwon H-J, Kim HS (2012). Signaling for synergistic activation of natural killer cells. Immune Netw.

[62] Tassi I, Presti R, Kim S, Yokoyama WM, Gilfillan S, Colonna M (2005). Phospholipase C-gamma 2 is a critical signaling mediator for murine NK cell activating receptors. J Immunol.

