# Supplementary material for: TREM2 promotes natural killer cell development in CD3−CD122+NK1.1+ pNK cells
Source: BMC Immunol. 2021 May 12;22:30. doi: 10.1186/s12865-021-00420-0 (PMC8114489; doi:10.1186/s12865-021-00420-0)
Supplement: Supplementary file 1 — Additional file 1: Fig. S1. The number of NK1.1+ cells, from TREM2-TG mice, expressing NK cell receptors is higher than in WT mice. Graphs show the absolute number of cells that express the NK cell receptors in the spleen, BM, and liver of WT (opened bar) and TREM2-TG mice (solid bar). Data are shown as mean ± SED of three independent experiments. *P < 0.05, **P < 0.01, and ***P < 0.001 by Student’s t-test. Fig. S2. TREM2-TG mice show enhanced NK cell cytotoxicity. (A) Real-time qPCR analysis to determine expression of the TREM2, Ifng, Prf1, and Gzmb (granzyme B) mRNAs in splenic NK1.1+ cells of WT or TREM2-TG mice. (B) LDH assay to measure cytotoxicity of NK1.1+ cells purified from splenocytes harvested from WT (open square) and TREM2-TG (solid circle) mice. Three independent experiments were performed (A-B). *P < 0.05, **P < 0.01, and ***P < 0.001 by Student’s t-test. Fig. S3. CD4+T cell, CD8+T cell, and B220+ B cell frequency and absolute number in WT and TREM2-TG mice are similar. Percentage (A) and absolute number (B) of CD4, CD8, and B cells in spleen of WT and TREM2-TG mice determined by flow cytometry (N = 5). Three independent experiments were performed, but no significant differences were observed between WT and TG mice group. Fig. S4. Inhibition of TREM2 signaling reduces the NK cell pool in vivo. Representative flow cytometry plots of expression of NK cell-specific receptors (NKG2A/C/E, Ly49C/F/H/I, and Ly49D) on surface of cells isolated from the spleen, BM, and liver of WT mice injected (i.p.) with 100 μg of TREM2-Ig or hu-Ig (control) twice per week for 4 weeks. Fig. S5. TREM2 signaling enhances NK cell-related gene expression in differentiated NK cells in vitro. Quantitative real-time PCR analysis of NK cell-associated genes using mRNA isolated from mNK cells derived from WT or TREM2-TG HSCs. Data are shown as mean ± SED of three independent experiments. *P < 0.05, **P < 0.01, ***P < 0.001 vs. WT+ hu-Ig, and †P < 0.05, ††P < 0.01, †††P < 0.01 vs. TG [file 12865_2021_420_MOESM1_ESM.docx]

TREM2 promotes natural killer cell development in CD3^-^CD122^+^NK1.1^+^ pNK cells

Hwa-Youn Lee^1,a^, Eun-Hee Lee^2,a^, Jawoon Yi^3^, Kon-Young Ji^4^, Su-Man Kim^3^, Ha-Rim Choi^5^, Su-Min Yee^3^, Hyung-Sik Kang^3,*^, and Eun-Mi Kim^6,*^

^1^ Chemicals Registration & Evaluation Team, National Institute of Environmental Research, Hwangyeong-ro 42, Seo-gu, Incheon 22689, Korea

^2^ Medical Device Development Center, Daegu-Gyeongbuk Medical Innovation Foundation, 80 Cheombok-ro, Dong-gu, Daegu 41061, Korea

^3^ School of Biological Sciences and technology, Chonnam National University, 77 Yongbong-ro, Buk-gu, Gwangju 61186, Korea

^4^ Herbal Medicine Research Division, Korea Institute of Oriental Medicine, 461-24 Jeonmin-dong, Yuseong-gu, Daejeon 34054, Korea

^5^ Department of Nursing, Nambu University, 23 Chumdan Jungang-ro, Gwangsan-gu, Gwangju 62271, Korea

^6^ Department of Predictive Toxicology, Korea Institute of Toxicology, 141 Gajeong-ro, Yuseong-gu, Daejeon 34114, Korea

***Corresponding authors:**

HS Kang, Ph.D., School of Biological Sciences and Technology, Chonnam National University, 77 Youngbong-ro, Buk-gu, Gwangju 61186, Republic of Korea**;** Email: kanghs@jnu.ac.kr; Tel: +82-62-530-2195.

Eun-Mi Kim, Ph.D., Department of Predictive Toxicology, Korea Institute of Toxicology, 141 Gajeong-ro, Yuseong-gu, Daejeon 34114, Republic of Korea; Email: eunmi.kim@kitox.re.kr; Tel: +82-42-610-8263

**This file includes:**

Figures S1 to S9


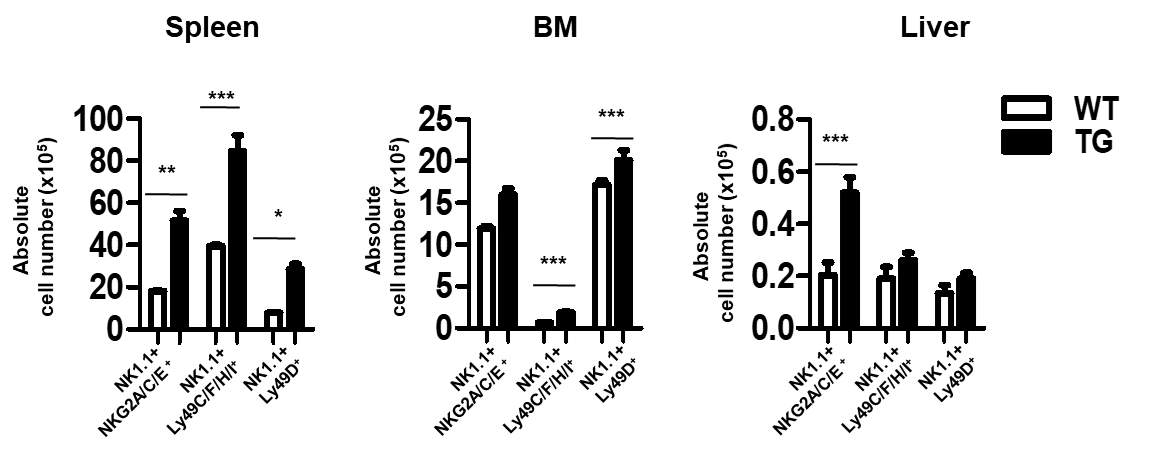


**Fig. S1.** **The number of NK1.1^+^ cells, from TREM2-TG mice, expressing NK cell receptors is higher than in WT mice.** Graphs show the absolute number of cells that express the NK cell receptors in the spleen, BM, and liver of WT (opened bar) and TREM2-TG mice (solid bar). Data are shown as mean ± SED of three independent experiments. *P < 0.05, **P <0.01, and ***P < 0.001 by Student’s t-test.

**Liver**

**NK1.1+**

**NKG2A/C/E ^+^**

**NK1.1+**

**Ly49C/F/H/I^+^**

**NK1.1+**

**Ly49D^+^**

******

*******

*****

*******

*******

*******

**NK1.1+**

**NKG2A/C/E ^+^**

**NK1.1+**

**Ly49C/F/H/I^+^**

**NK1.1+**

**Ly49D^+^**

**NK1.1+**

**NKG2A/C/E ^+^**

**NK1.1+**

**Ly49C/F/H/I^+^**

**NK1.1+**

**Ly49D^+^**

**Absolute**

**cell number (x10^5^)**

**Absolute**

**cell number (x10^5^)**

**Absolute**

**cell number (x10^5^)**

**Spleen**

**BM**


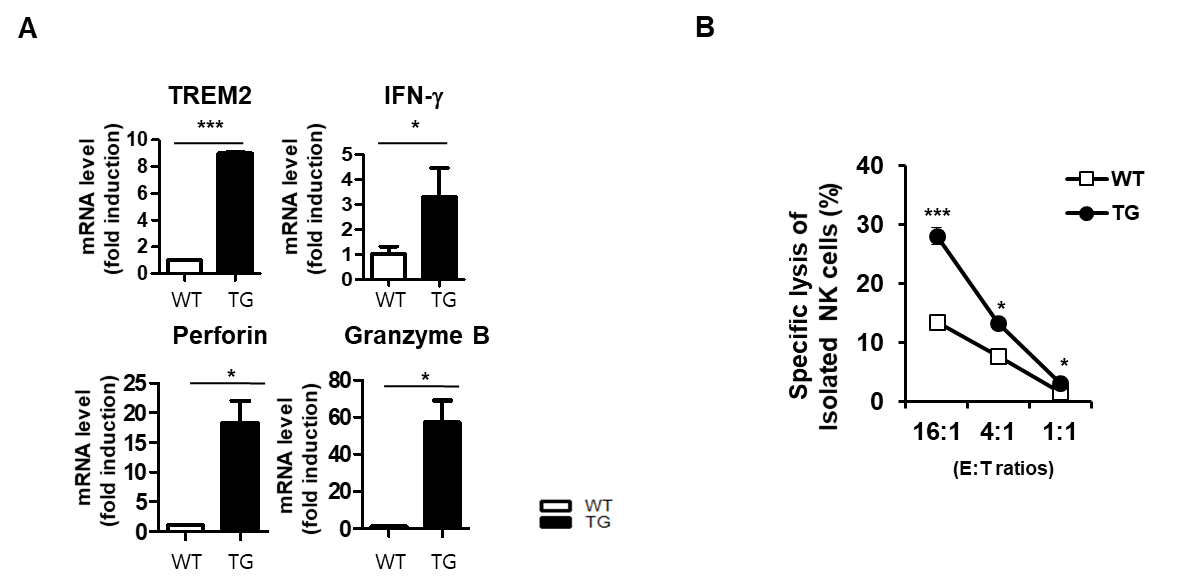


**Fig. S2. TREM2-TG mice show enhanced NK cell cytotoxicity.** (A) Real-time qPCR analysis to determine expression of the *TREM2*, *Ifng*, *Prf1*, and *G*zmb (granzyme B) mRNAs in splenic NK1.1^+^ cells of WT or TREM2-TG mice. (B) LDH assay to measure cytotoxicity of NK1.1^+^ cells purified from splenocytes harvested from WT (open square) and TREM2-TG (solid circle) mice. Three independent experiments were performed (A-B). *P < 0.05, **P <0.01, and ***P < 0.001 by Student’s t-test.
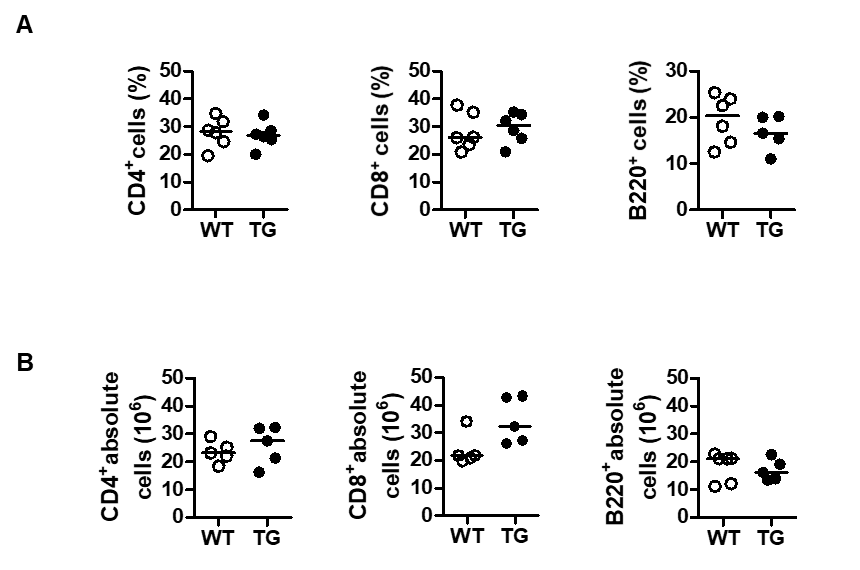


**Fig. S3. CD4^+^T cell, CD8^+^T cell, and B220^+^ B cell frequency and absolute number in WT and TREM2-TG mice are similar.** Percentage (A) and absolute number (B) of CD4, CD8, and B cells in spleen of WT and TREM2-TG mice determined by flow cytometry (N=5). Three independent experiments were performed, but no significant differences were observed between WT and TG mice group.


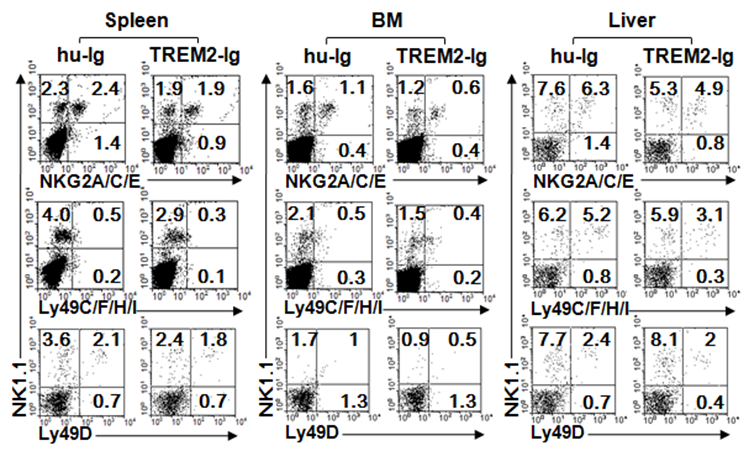


**Fig. S4. Inhibition of TREM2 signaling reduces the NK cell pool *in vivo*.** Representative flow cytometry plots of expression of NK cell-specific receptors (NKG2A/C/E, Ly49C/F/H/I, and Ly49D) on surface of cells isolated from the spleen, BM, and liver of WT mice injected (i.p.) with 100 µg of TREM2-Ig or hu-Ig (control) twice per week for 4 weeks.


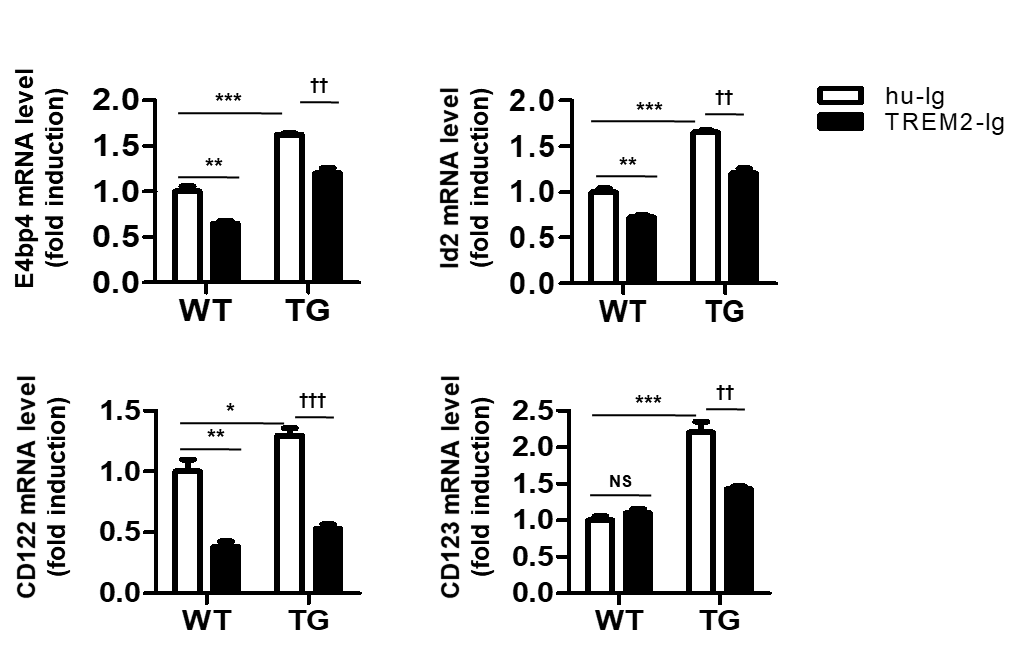


**Fig. S5.** **TREM2 signaling enhances NK cell-related gene expression in differentiated NK cells *in vitro*.** Quantitative real-time PCR analysis of NK cell-associated genes using mRNA isolated from mNK cells derived from WT or TREM2-TG HSCs. Data are shown as mean ± SED of three independent experiments. *P < 0.05, **P < 0.01, ***P < 0.001 vs. WT+ hu-Ig, and †P < 0.05, ††P < 0.01, †††P < 0.01 vs. TG+hu-Ig, based on two-way ANOVA with Bonferroni post-hoc test.


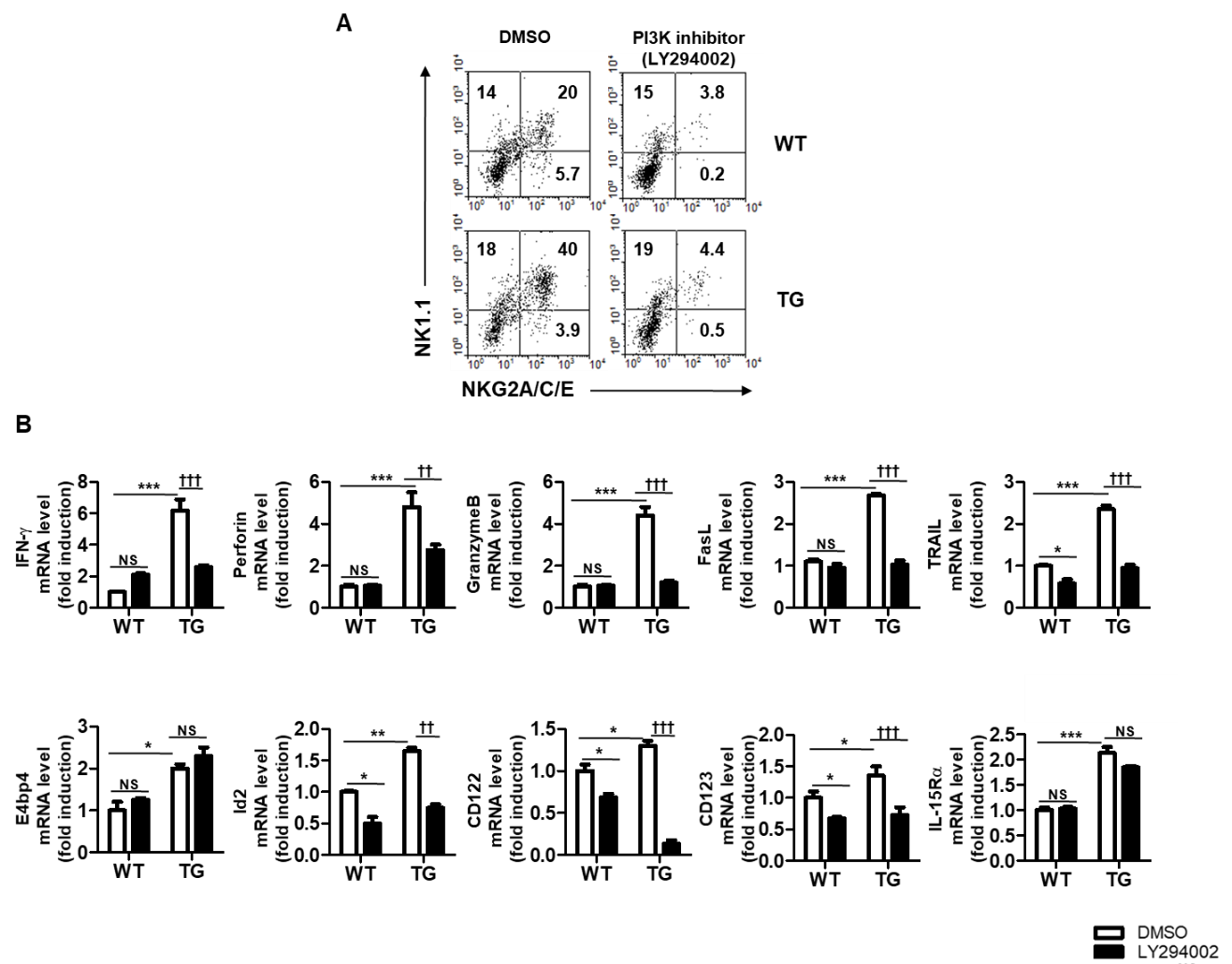


**Fig. S6. The development and cytotoxicity of NK cells is regulated by TREM2 via PI3K signaling.** (A) Representative flow cytometry plots showing the relative ratio of mNK cells developed *in vitro* from pNK cells of WT-HSCs and TREM2-HSCs treated with DMSO or LY294002 (1 µM). (B) Quantitative real-time PCR analysis of the indicated genes using mRNA isolated from mNK cells differentiated from WT-HSCs or TREM2-TG-HSCs treated with DMSO (opened bar) or LY294002 (solid bar). *P < 0.05, **P < 0.01, ***P < 0.001 vs. WT+ DMSO, and †P < 0.05, ††P < 0.01 vs. TG+DMSO, based on two-way ANOVA with Bonferroni post-hoc test.


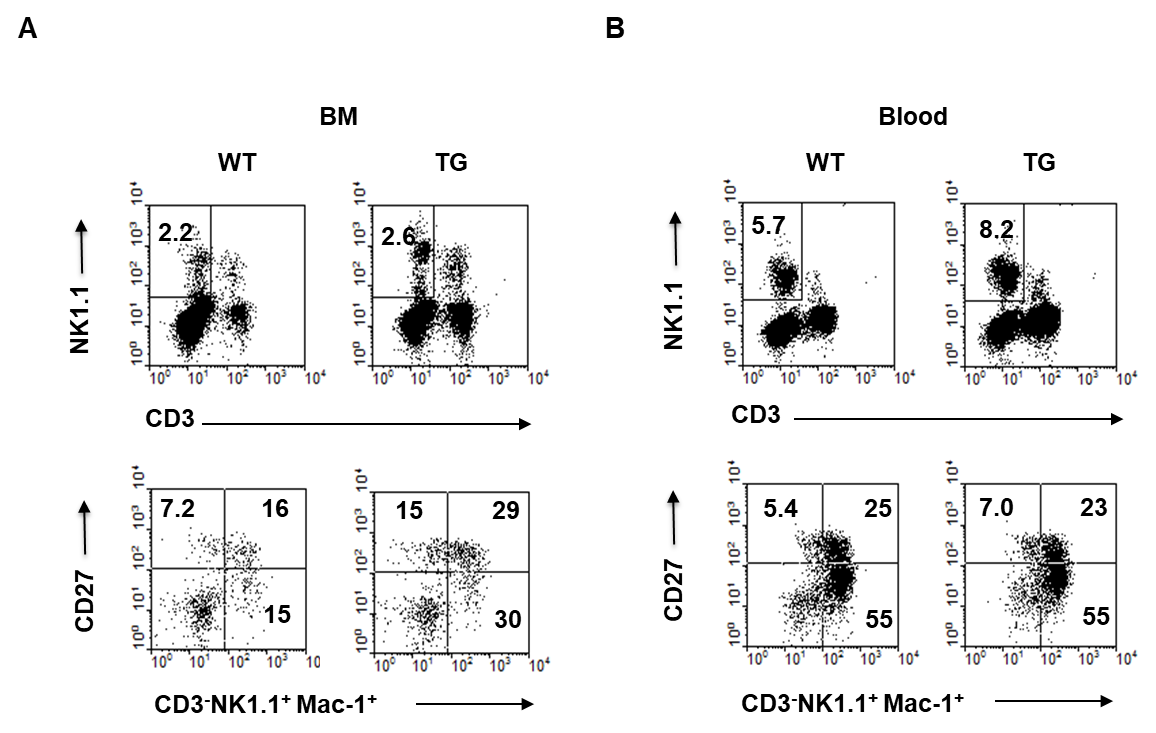


**Fig. S7. The CD3^-^NK1.1^+^Mac-1^+^CD27^+^ NK cell subset is reduced in peripheral blood of TREM2-K/O mice.** Representative flow cytometry plots show the NK cell subsets (CD3^-^NK1.1^+^Mac-1^low^CD27^high^, CD3^-^NK1.1^+^Mac-1^high^ CD27_high_, CD3^-^NK1.1^+^Mac-1^high^ CD27^low^, and CD3^-^NK1.1^+^Mac-1^low^CD27^high^) in the peripheral blood (A) or BM (B) of WT and TREM2-TG mice. Three independent experiments were performed.


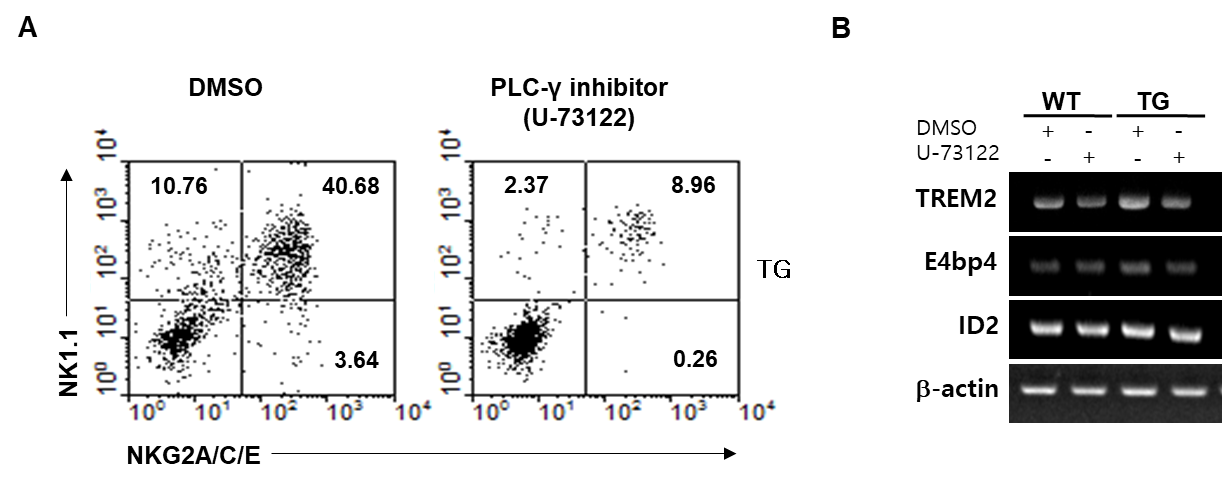


**Fig. S8. TREM2 regulates NK cell differentiation via PLC-γ signaling pathway.** (A) Representative flow cytometry analysis showing NK1.1 and NKG2A/C/E expression on the differentiated NK cells derived from TG-HSCs treated with DMSO (control) or U-73122 (PLC-γ inhibitor) during pNK to mNK cell differentiation stage. (B) RT-PCR analysis of the indicated genes on mNK cells differentiated from WT-HSCs or TREM2 TG-HSCs treated with DMSO (control) or U-73122 (PLC-γ inhibitor). β-actin (housekeeping gene) was used as a control. The cropped images from the different parts of the gel were displayed. The full-length gels are presented in Fig. S9.


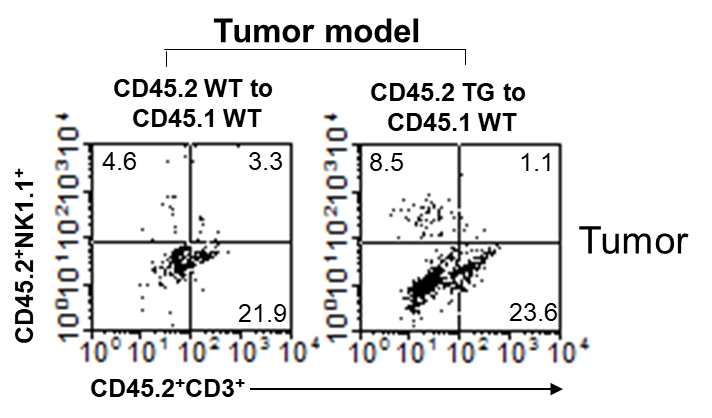


**Fig. S9. The CD45.2^+^NK1.1^+^ cells persist in the tumor site in the recipient mice that received TREM2-TG-BM transplantation.** Representative flow cytometry analysis of the expression of CD45.2^+^ NK1.1^+^ cells derived from TREM2-TG-HSCs in the tumor model (N=3). Data from day 41 after the 4 weeks adaptive transplantation following period.


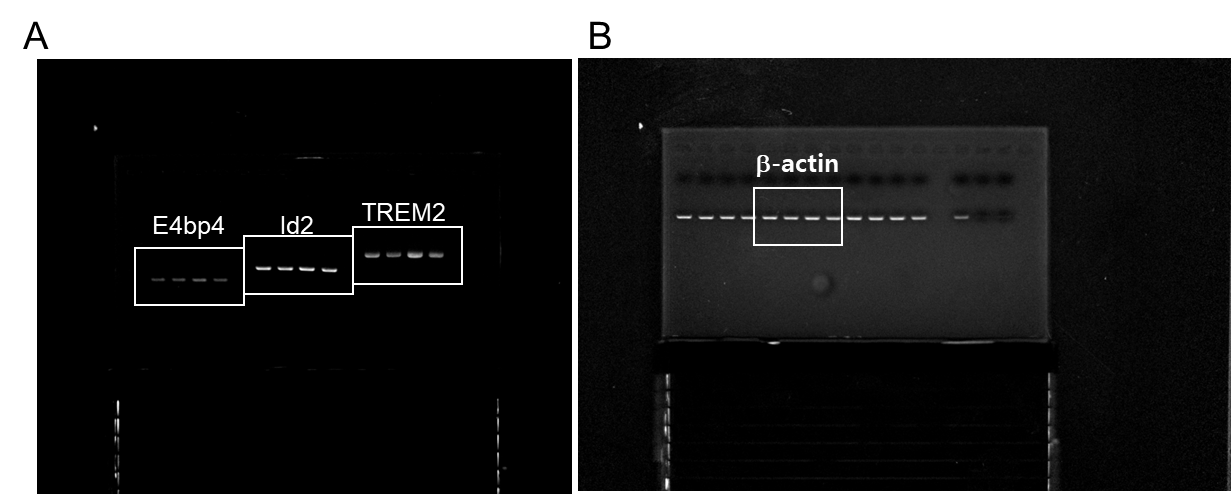


**Fig. S10. Full-length gel images of cropped gels.** (A) Full-length gel corresponding to Fig. S8B; E4bp4 (left), Id2 (middle), TREM2 (right). (B) Full-length gel corresponding to Fig. S8B, *β*-actin.
